# Propranolol and survival from breast cancer: a pooled analysis of European breast cancer cohorts

**DOI:** 10.1186/s13058-016-0782-5

**Published:** 2016-12-01

**Authors:** Chris R. Cardwell, Anton Pottegård, Evelien Vaes, Hans Garmo, Liam J. Murray, Chris Brown, Pauline A. J. Vissers, Michael O’Rorke, Kala Visvanathan, Deirdre Cronin-Fenton, Harlinde De Schutter, Mats Lambe, Des G. Powe, Myrthe P. P. van Herk-Sukel, Anna Gavin, Søren Friis, Linda Sharp, Kathleen Bennett

**Affiliations:** 1 Institute of Clinical Sciences Block B, Centre for Public Health, Queen’s University Belfast, Royal Victoria Hospital, Belfast, BT12 6BA UK; 2 Department of Public Health, University of South Denmark, Odense, Denmark; 3 Research Department, Belgian Cancer Registry, Brussels, Belgium; 4 Division of Cancer Studies, Cancer Epidemiology Unit, King’s College London, London, UK; 5 Regional Cancer Centre Uppsala-Örebro, Uppsala, Sweden; 6 National Cancer Registry Ireland, Cork, Ireland; 7 Netherlands Comprehensive Cancer Organisation, Utrecht, The Netherlands; 8 Johns Hopkins University Bloomberg School of Public Health and School of Medicine, Baltimore, USA; 9 Department of Clinical Epidemiology, Aarhus University, Aarhus, Denmark; 10 Department of Medical Epidemiology and Biostatistics, Karolinska Institutet, Stockholm, Sweden; 11 Department of Cellular Pathology, Queens Medical Centre, NUH, Nottingham, UK; 12 PHARMO Institute for Drug Outcomes Research, Utrecht, The Netherlands; 13 Northern Ireland Cancer Registry, Queen’s University Belfast, Belfast, UK; 14 Danish Cancer Society Research Center, Danish Cancer Society, Copenhagen, Denmark; 15 Institute of Health and Society, Newcastle University, Newcastle upon Tyne, UK; 16 Department of Pharmacology & Therapeutics, Trinity College Dublin, Dublin, Ireland

**Keywords:** Breast cancer, Mortality, Beta-blocker, Cohort

## Abstract

**Background:**

Preclinical studies have demonstrated that propranolol inhibits several pathways involved in breast cancer progression and metastasis. We investigated whether breast cancer patients who used propranolol, or other non-selective beta-blockers, had reduced breast cancer-specific or all-cause mortality in eight European cohorts.

**Methods:**

Incident breast cancer patients were identified from eight cancer registries and compiled through the European Cancer Pharmacoepidemiology Network. Propranolol and non-selective beta-blocker use was ascertained for each patient. Breast cancer-specific and all-cause mortality were available for five and eight cohorts, respectively. Cox regression models were used to calculate hazard ratios (HR) and 95% confidence intervals (CIs) for cancer-specific and all-cause mortality by propranolol and non-selective beta-blocker use. HRs were pooled across cohorts using meta-analysis techniques. Dose–response analyses by number of prescriptions were also performed. Analyses were repeated investigating propranolol use before cancer diagnosis.

**Results:**

The combined study population included 55,252 and 133,251 breast cancer patients in the analysis of breast cancer-specific and all-cause mortality respectively. Overall, there was no association between propranolol use after diagnosis of breast cancer and breast cancer-specific or all-cause mortality (fully adjusted HR = 0.94, 95% CI, 0.77, 1.16 and HR = 1.09, 95% CI, 0.93, 1.28, respectively). There was little evidence of a dose–response relationship. There was also no association between propranolol use before breast cancer diagnosis and breast cancer-specific or all-cause mortality (fully adjusted HR = 1.03, 95% CI, 0.86, 1.22 and HR = 1.02, 95% CI, 0.94, 1.10, respectively). Similar null associations were observed for non-selective beta-blockers.

**Conclusions:**

In this large pooled analysis of breast cancer patients, use of propranolol or non-selective beta-blockers was not associated with improved survival.

## Background

Beta-blockers, used for heart disease and hypertension [1], act by inhibiting beta-adrenergic receptors. Recent studies have shown that breast cancer tissue expresses beta-adrenergic receptors [2], particularly type 2 beta-adrenergic receptors [3]. Numerous in-vitro studies have demonstrated that beta-blockers can disrupt migratory activity and inhibit angiogenesis of cancer cells [4, 5]. In particular, propranolol appears to have potent anti-migratory and anti-angiogenic properties as demonstrated in cancer cell lines and animal models [4–10]. This preclinical evidence has led to calls for randomised controlled trials of propranolol as adjuvant therapy in breast cancer patients [11]; however, although early phase trials are underway [12, 13], phase 3 trials have not been conducted to date.

Only three observational studies have previously investigated the association between propranolol use and breast cancer outcomes. In 2011, an Irish study observed an 80% reduction in breast cancer-specific mortality among patients who used propranolol in the year prior to diagnosis [14]. No association was observed between propranolol use after diagnosis and breast cancer-specific mortality in an English study [15] or between propranolol use after diagnosis and breast cancer recurrence in a Danish study [16]. However, these studies had limited power because of the small numbers of breast cancer patients using propranolol, reflecting the low prevalence of propranolol use which in each study was under 5% [14–16]. Therefore, a need remains to further investigate propranolol (and other non-selective beta-blockers) and survival in breast cancer patients to inform the decision on whether to conduct large phase 3 randomised controlled trials of propranolol as adjuvant cancer therapy in breast cancer patients.

Consequently, utilising the European Cancer Pharmacoepidemiology Network [17], we conducted a pooled analysis of eight cohorts of breast cancer patients from across Europe to examine whether use of propranolol (or other non-selective beta-blockers) is associated with improved breast cancer-specific and all-cause mortality.

## Methods

### Data sources

Eight cohorts of breast cancer patients from across Europe (including Belgium, Denmark, England, the Netherlands, Northern Ireland, Republic of Ireland, Scotland and Sweden) were compiled through the European Cancer Pharmacoepidemiology Network [17]. Characteristics of these cohorts are presented in Table 1. The association between propranolol use and cancer mortality was examined previously within the English cohort [15] (although using a nested case–control design) and the Republic of Ireland cohort [14] (although this analysis did not investigate propranolol use after diagnosis, had shorter follow-up and had substantially fewer cases). Cancer recurrence was investigated previously in the Danish cohort [16] (although the earlier analysis was based on fewer than 20% of the breast cancer patients included in the present analysis and did not investigate mortality). Previous studies have reported detailed descriptions of the medication data available and/or linkages available in the cohorts from Denmark [18–20], England [21], the Netherlands [22, 23], Northern Ireland [24], Belgium [25], Republic of Ireland [26], Scotland [27] and Sweden [28, 29].

**Table 1:** Characteristics of the included cohorts

Country	Breast cancer		Medication data	Mortality data					Additional^a^ confounders								
	source	Diagnosis years	source	source	End f-up	Mean f-up (years)	Max f-up (years)	BC specific	Grade	Surg	Radio	Chemo	Tam	AI	Asp/statin	HRT	Comorbidity
Belgium	Belgian Cancer Registry	2007–2009	Intermutualistic Agency (health insurance records)	Kruispuntbank van de Sociale Zekerheid (social security records)	2014	6	8	No	Yes	Yes	Yes	Yes	Yes	Yes	Yes	Yes	No
Denmark	Danish Cancer Registry^b^	2000–2012	Danish National Prescription Registry	Danish Civil Registration System	2012	6	13	No	Yes	Yes	Yes	Yes	Yes	Yes	Yes	Yes	Yes^c^
England (UK)	National Cancer Data Repository	1998–2007	CPRD (GP records)	Office of National Statistics	2011	6	12	Yes	Yes	Yes	Yes	Yes	Yes	Yes	Yes	No	Yes^d^
The Netherlands	Eindhoven Cancer Registry	1999–2011	PHARMO (pharmacy records)	Central Bureau of Genealogy	2012	6	13	No	Yes	Yes	Yes	Yes	Yes	Yes	Yes	Yes	Yes^g^
Northern Ireland (UK)	Northern Ireland Cancer Registry	2009–2010	NIEPD (electronic dispensing database)	General Register Office for Northern Ireland	2013	4	5	Yes	No	Yes	N	Yes	Yes	Yes	Yes	No	No
Republic of Ireland	National Cancer Registry Ireland	2001–2010	GMS^e^ (electronic prescribing database)	Central Statistics Office (Death Certificates)	2012	5	11	Yes	Yes	Yes	Yes	Yes	Yes	Yes	Yes	Yes	Yes^f^
Scotland (UK)	Scottish Cancer Registry	2009–2012	Prescribing Information System (electronic dispensing database)	National Records of Scotland Death Records	2015	4	6	Yes	Yes	Yes	Yes	Yes	Yes	Yes	Yes	No	Yes^c^
Sweden	Regional cancer registries in Norra, Uppsala/Örebro Stockholm/Gotland	2007–2012	The Prescribed Drug Register	Cause of death registry	2012	4	6	Yes	Yes	Yes	Yes	Yes	Yes	Yes	Yes	Yes	Yes^c^

^a^In fully adjusted analyses (presented in Tables 2 and 3), the model contains age at diagnosis, year of diagnosis, stage and the variables shown

^b^Only including stages 1–3

^c^Based on hospital admissions

^d^Based on GP diagnosis codes

^e^GMS includes eligible patients based upon means test and age (all patients over 70 years old are included)

^f^Based on RxRisk comorbidity score

^g^From cancer registry records

*AI* aromatase inhibitors, *asp* aspirin, *BC* breast cancer, *chemo* chemotherapy, *CPRD* clinical practice research datalink, *f-up* follow-up duration from diagnosis to death or censoring, *GMS* General Medical Services scheme, *GP* general practitioner, *HRT* hormone replacement therapy, *max* maximum, *NIEPD* Northern Ireland Electronic Prescribing Database, *radio* radiotherapy, *surg* surgery, *tam* tamoxifen

### Inclusion criteria

All cohorts identified incident invasive breast cancer patients from cancer registries. The year of diagnosis for included breast cancer patients varied across the cohorts from 1998 to 2012. Patients with other invasive cancer diagnoses (apart from non-melanoma skin cancer) prior to their breast cancer diagnosis were excluded.

### Exposure

Propranolol and all non-selective beta-blocker use (including propranolol, sotalol, timolol, nadolol, carvedilol, pindolol, oxprenolol and labetolol) was ascertained from electronic dispensing records in five cohorts, GP prescribing records in two cohorts and health insurance records in one cohort (see Table 1).

### Outcome

In seven of the cohorts, mortality was ascertained from national death records; social security records were used in one cohort (see Table 1). Breast cancer-specific mortality was defined as breast cancer being the underlying cause of death and was available in five cohorts. All-cause mortality was available in all cohorts.

### Covariates

The covariates available varied between cohorts and were obtained from a number of sources including cancer registries, hospital admissions, prescriptions, GPs and health insurance databases (see Table 1). The covariates recorded included: age, year of cancer diagnosis, stage, grade, cancer treatment within the first 6 months after diagnosis (including information on cancer-directed surgery, chemotherapy, radiotherapy), medication use (including tamoxifen, aromatase inhibitors, hormone replacement therapy (prior to diagnosis), aspirin [30], statins [31]) and comorbidities prior to diagnosis. Cancer-directed surgery, chemotherapy and radiotherapy were taken from cancer registry records, apart from in Belgium where insurance claims were used and in Denmark where Patient Registry records were used. Comorbidities, largely including those in the Charlson comorbidity index [32], were taken from hospital admission records in Denmark, Scotland and Sweden, from GP records in England and from cancer registry records in the Netherlands. In the cohorts from the Netherlands, Denmark and England, adjustments for comorbidity were made for cerebrovascular disease, chronic pulmonary disease, congestive heart disease, diabetes, myocardial infarction, peptic ulcer disease, peripheral vascular disease and renal disease. In Sweden additional adjustments were made for liver disease and in Scotland additional adjustments were made for liver disease and diabetes complications. In the Republic of Ireland cohort, comorbidity information was based upon prescribing information using the RxRisk score [33]. Oestrogen use was based upon HRT use any time prior to diagnosis in the Netherlands, HRT or oral contraceptive use in the year prior to diagnosis in Denmark or HRT use in the year prior to diagnosis in Sweden, the Republic of Ireland, Belgium and Scotland. Tamoxifen and aromatase inhibitor use was obtained from prescription records, except in Denmark were a single more complete endocrine therapy variable, based upon Patient Registry records, was used instead.

### Statistical analysis

We performed a two-stage analysis procedure allowing for adjustment of covariates which were not uniformly defined, coded or available across cohorts [34]. In the main analysis of medication use after diagnosis, the patients in each cohort were followed from 1 year after breast cancer diagnosis to death or end of follow-up, whichever was sooner. Patients who had died in the first year after breast cancer diagnosis (or who had less than 1 year of follow-up) were excluded because it seemed unlikely that propranolol use after diagnosis could reduce mortality within such a short period. In the main analysis, propranolol use was modelled as a time-varying covariate to avoid immortal time bias [35]; that is, patients were initially considered non-users and then became users a lag of 1 year after their first propranolol prescription. The use of a lag period is recommended in studies of medication use and cancer survival [36] because prescriptions filled shortly prior to death may reflect end-of-life treatment. In dose–response analyses, one propranolol prescription corresponded to 1 month of use, except for Denmark where one prescription corresponded to 3 months of use (based on the average duration of propranolol prescriptions in Denmark). In dose–response analyses, an individual was considered a non-user prior to 1 year after first medication usage, a user of 0–1 year for prescriptions from 1 year after first prescription to 1 year of prescriptions (considered four prescriptions in Denmark and 12 prescriptions in all other countries) and a greater user after this time. Time-dependent Cox regression models were used to calculate hazard ratios (HRs) and 95% confidence intervals (CIs) for breast cancer-specific death in propranolol users compared with propranolol non-users. An unadjusted analysis was first conducted, then an adjusted analysis (including just the covariates age and year of diagnosis in the model, which were available in all cohorts) and finally a fully adjusted analysis was conducted (including all covariates available within each cohort, as presented in Table 1, in the model). The summary HRs and standard errors (SEs) from the eight cohorts were combined using random effects models to calculate pooled HRs [37] and the consistency of HRs was investigated using chi-squared tests for heterogeneity and *I* ^2^ statistics [38]. The analyses were repeated for all-cause mortality. Analyses were then repeated comparing all non-selective beta-blockers users with non-selective beta-blocker non-users and comparing all beta-blocker users with beta-blocker non-users. A sensitivity analysis was conducted restricting the cohorts to patients with stage 1–3 breast cancer, because it is plausible that the effect might be most evident in those without advanced disease.

We performed two predefined secondary analyses. First, avoiding immortal time bias without requiring complex analyses [39], we compared users of propranolol (and separately users of non-selective beta-blockers) within the first year after breast cancer diagnosis with non-users within the same period, and started follow-up 1 year after breast cancer diagnosis. Second, to investigate the potential impact of propranolol use earlier in the process of cancer development, we performed a separate analysis of medication use before breast cancer diagnosis comparing time to death in propranolol (and non-selective beta-blockers) users with non-users in the year prior to diagnosis, restricted to individuals with at least 1 year of medication records prior to diagnosis. In analysis of pre-diagnostic medication use, patients who died in the first year after diagnosis (who had follow-up of less than 1 year) were not excluded.

## Results

### Patient cohorts

The pooled analysis for breast-cancer specific and all-cause mortality comprised 55,252 newly diagnosed breast cancer patients (in whom there were 5419 breast cancer-specific deaths and 9295 all-cause deaths) and 133,251 newly diagnosed breast cancer patients (in whom there were 25,472 all-cause deaths), respectively. The maximum follow-up in each cohort after diagnosis of breast cancer ranged from 5 to 13 years (see Table 1).

### Patient characteristics

Patient characteristics by propranolol (and non-selective beta-blocker) use in the first year after diagnosis are presented in Table 2. Propranolol users were slightly more likely to have an earlier year of breast cancer diagnosis. Age, stage, grade and cancer treatments were generally similar by propranolol use. There was a higher use of hormone antagonists (tamoxifen 39% versus 30% and aromatase inhibitors 26% versus 23%, respectively) in propranolol users versus non-users, but use of other medications was similar.

**Table 2:** Characteristics of breast cancer patients by propranolol and non-selective beta-blocker use in the year after diagnosis

	Propranolol in year after diagnosis^a^				Non-selective beta-blocker in year after diagnosis^a^			
Characteristic	Users		Non-users^b^		Users		Non-users^c^	
	*n*	%	*n*	%	*n*	%	*n*	%
Country								
Belgium	984	35.7	25,021	19.2	2131	42.1	23,874	18.6
Denmark	615	22.3	44,049	33.8	1198	23.6	43,466	33.9
England	212	7.7	9602	7.4	299	5.9	9515	7.4
The Netherlands	78	2.8	7252	5.6	203	4.0	7130	5.6
Northern Ireland	70	2.5	2106	1.6	82	1.6	2094	1.6
Republic of Ireland	142	5.2	9720	7.4	250	4.9	9612	7.5
Scotland	400	14.5	14,740	11.3	468	9.2	14,672	11.4
Sweden	255	9.3	18,005	13.8	436	8.6	17,824	13.9
Year of cancer diagnosis								
1995–1999	61	2.2	2495	1.9	84	1.7	2472	1.9
2000–2004	480	17.4	27,621	21.2	832	16.4	27,269	21.3
2005–2009	1 572	57.0	66,186	50.7	3219	63.5	64,542	50.3
2010–2014	643	23.3	34,193	26.2	932	18.4	33,904	26.4
Age at cancer diagnosis								
<40	97	3.5	5591	4.3	110	2.2	5578	4.4
40–49	444	16.1	19,688	15.1	540	10.7	19,592	15.3
50–59	730	26.5	31,297	24.0	1031	20.3	30,996	24.2
60–69	768	27.9	35,528	27.2	1419	28.0	34,877	27.2
70–79	491	17.8	23,488	18.0	1235	24.4	22,744	17.7
80–89	200	7.3	12,974	9.9	647	12.8	12,527	9.8
≥90	26	0.9	1929	1.5	82	1.6	1873	1.5
Stage								
1	965	35.0	49,458	37.9	1735	34.2	48,669	38.0
2	927	33.6	41,797	32.0	1718	33.9	41,006	32.0
3	284	10.3	9073	7.0	510	10.1	8847	6.9
4	138	5.0	5086	3.9	258	5.1	4966	3.9
Missing	442	16.0	25,081	19.2	843	16.6	24,699	19.3
Grade								
Well differentiated	365	17.2	16,827	19.7	662	17.2	16,530	19.7
Moderately differentiated	885	41.6	34,600	40.5	1543	40.2	33,942	40.5
Poorly differentiated	639	30.0	22,964	26.9	1109	28.9	22,494	26.8
Missing	239	11.2	11,115	13.0	529	13.8	10,825	12.9
Cancer treatment within 6 months of cancer diagnosis								
Surgery	2 425	88.0	114,271	87.6	4387	86.6	112,309	87.7
Chemotherapy	1 034	37.6	46,018	35.3	1608	31.8	45,444	35.5
Radiotherapy^d^	1 463	54.6	68,817	53.7	2649	53.2	67,631	53.7
Medication use in year after diagnosis								
Aromatase inhibitor^e^	724	33.8	29,176	33.8	1459	37.7	28,441	33.6
Tamoxifen^e^	1 064	49.7	39,087	45.2	1765	45.6	38,386	45.3
Statin	532	19.3	22,245	17.0	1353	26.7	21,424	16.7
Low-dose aspirin	368	13.4	17,545	13.4	1097	21.6	16,816	13.1

^a^Restricted to breast cancer patients living more than 1 year after diagnosis

^b^Propranolol non-users in the year after diagnosis, but could have used other beta-blockers

^c^Non-selective beta-blocker non-users in the year after diagnosis, but could have used other beta-blockers

^d^Refers to radiotherapy within 6 months of breast cancer diagnosis, except in Belgium where radiotherapy was considered within 9 months

^e^Excluding Denmark because aromatase inhibitor and tamoxifen were not recorded separately

### Association between propranolol use after diagnosis and breast cancer-specific and all-cause mortality

**Fig. 1:**
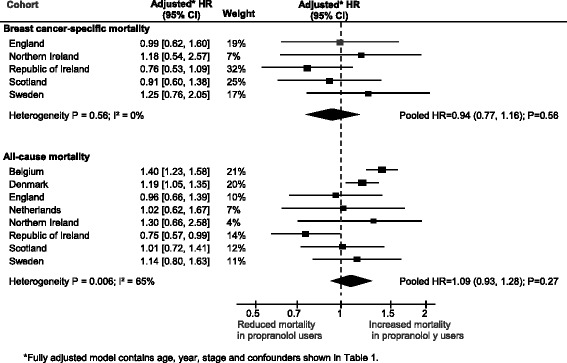
Association between propranolol and breast cancer-specific and all-cause mortality, by cohort. *Fully adjusted model contains age, year, stage and confounders presented in Table 1. *CI* confidence interval, *HR* hazard ratio

Overall 4746 breast cancer patients used propranolol at any time after diagnosis (1768 from Belgian, 1057 from Denmark, 419 from England, 151 from the Netherlands, 107 from Northern Ireland, 232 from the republic of Ireland, 629 from Scotland and 383 from Sweden). Table 3 and Fig. 1 present the findings from the main analysis. Overall, there was little difference in breast cancer-specific mortality or all-cause mortality in propranolol users compared with non-users after diagnosis (fully adjusted HR = 0.94, 95% CI, 0.77, 1.16 and HR = 1.09, 95% CI, 0.93, 1.28, respectively). The associations between propranolol and cancer-specific mortality were fairly consistent across cohorts (*I* ^2^ = 0% and heterogeneity *P* = 0.56), whereas the association varied more for all-cause mortality (*I* ^2^ = 65% and heterogeneity *P* = 0.006). On closer inspection (see Fig. 1) this heterogeneity was partly due to the Belgian estimate; once this was removed the pooled estimate was attenuated slightly (fully adjusted HR = 1.03, 95% CI, 0.88, 1.20) and the heterogeneity was reduced (*I* ^2^ = 39% and heterogeneity *P* = 0.02). There was little evidence of a dose–response association; compared with propranolol non-users, there was no association between use of more than 1 year of propranolol prescriptions and cancer-specific or all-cause mortality (fully adjusted HR = 0.93, 95% CI, 0.46, 1.90 and HR = 1.09, 95% CI, 0.85, 1.40, respectively). Similar null associations were observed for cancer-specific mortality when comparing users of non-selective beta-blockers with non-users of non-selective beta-blockers (see Table 3).

**Table 3:** Pooled analysis of the association between propranolol and non-selective beta-blocker use after breast cancer diagnosis and breast cancer-specific and all-cause mortality

Medication usage	Cancer-specific/all-cause mortality	All patients	Person-years	Unadjusted			Adjusted for age and year			Fully adjusted^a^		
				HR (95% CI)	*P*	Hetero *I* ^2^ (*P*)	HR (95% CI)	*P*	Hetero *I* ^2^ (*P*)	HR (95% CI)	*P*	Hetero *I* ^2^ (*P*)
Breast cancer-specific mortality												
Propranolol non-user	5291	53,482	176,723	1.00 (ref. cat.)			1.00 (ref. cat.)			1.00 (ref. cat.)		
Propranolol user^b^	128	1770	4989	0.92 (0.77, 1.10)	0.36	0% (0.75)	0.97 (0.82, 1.16)	0.77	0% (0.84)	0.94 (0.77, 1.16)	0.56	0% (0.56)
Propranolol prescriptions												
<1 year of prescriptions^c^	88	1217	3703	1.00 (0.79, 1.26)	0.98	22% (0.27)	1.09 (0.88, 1.35)	0.42	9% (0.35)	1.01 (0.80, 1.27)	0.96	0% (0.62)
≥1 year of prescriptions^c^	40	553	1286	0.82 (0.56, 1.21)	0.32	3% (0.38)	0.80 (0.54, 1.17)	0.25	2% (0.38)	0.93 (0.46, 1.90)	0.84	63% (0.04)
Non-selective bb non-user	5215	52,903	175,007	1.00 (ref. cat.)			1.00 (ref. cat.)			1.00 (ref. cat.)		
Non-selective bb user^b^	204	2349	6706	1.08 (0.94, 1.24)	0.31	0% (0.51)	1.07 (0.93, 1.23)	0.37	0% (0.92)	1.01 (0.85, 1.20)	0.90	0% (0.47)
Non-selective bb prescriptions												
<1 year of prescriptions^c^	145	1466	4555	1.13 (0.93, 1.37)	0.22	22% (0.27)	1.16 (0.99, 1.37)	0.07	0% (0.55)	1.10 (0.90, 1.34)	0.36	0% (0.70)
≥1 year of prescriptions^c^	59	883	2149	1.02 (0.78, 1.31)	0.91	0% (0.72)	0.92 (0.71, 1.19)	0.53	0% (0.83)	0.97 (0.63, 1.48)	0.88	46% (0.14)
All-cause mortality												
Propranolol non-user	24,654	128,505	554,765	1.00 (ref. cat.)			1.00 (ref. cat.)			1.00 (ref. cat.)		
Propranolol user^b^	818	4746	16,202	1.04 (0.86, 1.27)	0.68	82% (<0.01)	1.13 (0.93, 1.37)	0.21	81% (<0.01)	1.09 (0.93, 1.28)	0.27	65% (0.006)
Propranolol prescriptions												
<1 year of prescriptions^c^	548	3099	10,977	1.01 (0.78, 1.32)	0.92	85% (<0.01)	1.20 (0.93, 1.53)	0.16	83% (<0.01)	1.15 (0.95, 1.39)	0.16	62% (0.01)
≥1 year of prescriptions^c^	270	1647	5225	1.18 (0.97, 1.44)	0.10	45% (0.09)	1.10 (0.96, 1.26)	0.17	10% (0.35)	1.09 (0.85, 1.40)	0.48	55% (0.04)
Non-selective bb non-user	23,740	125,320	543,344	1.00 (ref. cat.)			1.00 (ref. cat.)			1.00 (ref. cat.)		
Non-selective bb user^b^	1732	7931	27,624	1.34 (1.14, 1.58)	0.001	87% (<0.01)	1.22 (1.09, 1.36)	0.001	70% (<0.01)	1.16 (1.02, 1.32)	0.02	71% (<0.01)
Non-selective bb prescriptions												
<1 year of prescriptions^c^	1012	4512	17,074	1.23 (0.98, 1.55)	0.08	89% (<0.01)	1.22 (1.04, 1.43)	0.02	75% (<0.01)	1.19 (1.04, 1.36)	0.01	54% (0.03)
≥1 year of prescriptions^c^	720	3419	10,549	1.67 (1.49, 1.87)	<0.001	39% (0.13)	1.30 (1.21, 1.40)	<0.001	0% (0.61)	1.23 (1.04, 1.45)	0.02	62% (0.02)

^a^Model contains age, year, stage and confounders presented in Table 1

^b^Medication use modelled as a time-varying covariate with an individual considered a non-user prior to 1 year after first medication usage and a user after this time, excludes deaths in the year after cancer diagnosis

^c^Medication use modelled as a time-varying covariate with an individual considered a non-user prior to 1 year after first medication usage, a user of 0–1 year of prescriptions from 1 year after first prescription to 1 year of prescriptions (considered four prescriptions in Denmark and 12 prescriptions in all other countries) and a greater user after this time, excludes deaths in the year after cancer diagnosis

*bb* beta-blocker, *CI* confidence interval, *HR* hazard ratio, *ref. cat.* reference category

### Secondary and sensitivity analyses

Secondary and sensitivity analyses are presented in Table 4. In sensitivity analyses restricting the cohorts to stage 1–3 breast cancer patients only, the associations between propranolol and cancer-specific and all-cause mortality was similar to those for the main analysis (see Table 4). In secondary analysis there was no evidence of an inverse association between any beta-blocker use after diagnosis and cancer-specific or all-cause mortality (fully adjusted HR = 1.07, 95% CI, 0.99, 1.16 and HR = 1.12, 95% CI, 1.05, 1.20, respectively). The secondary analysis based upon medication use in the first year after diagnosis also produced similar results for propranolol and cancer-specific and all-cause mortality (fully adjusted HR = 1.07, 95% CI, 0.72, 1.60 and HR = 1.04, 95% CI, 0.89, 1.21, respectively).

**Table 4:** Secondary and sensitivity analyses for pooled analysis of the association between propranolol and non-selective beta-blocker use and breast cancer-specific and all-cause mortality

Medication usage	Deaths	Patients	Person-years	Unadjusted			Fully adjusted		
				HR (95% CI)	*P*	Hetero *I* ^2^ (*P*)	Adjusted HR (95% CI)	*P*	Hetero *I* ^2^ (*P*)
Breast cancer-specific mortality									
Medication use after diagnosis									
Main time-varying covariate analysis in stage 1–3 breast cancer patients									
Propranolol in stages 1–3	3389	44,376	112,450	1.02 (0.82, 1.26)	0.88	0% (0.84)	1.06 (0.85, 1.33)	0.62	0% (0.60)
Main time-varying covariate analysis in all breast cancer patients									
Any beta-blocker	5419	55,252	181,714	1.25 (1.11, 1.40)	<0.001	63% (0.03)	1.07 (0.99, 1.16)	0.10	0% (0.82)
Analysis based upon use in year after diagnosis^a^									
Propranolol	5426	55,252	181,959	0.94 (0.72, 1.21)	0.61	35% (0.19)	1.07 (0.72, 1.60)	0.72	65% (0.02)
Non-selective beta-blocker	5426	55,252	181,959	1.10 (0.87, 1.39)	0.43	51% (0.09)	1.15 (0.85, 1.56)	0.35	60% (0.04)
Medication use before diagnosis^b^									
Propranolol	6883	53,870	215,978	0.97 (0.82, 1.15)	0.73	0% (0.51)	1.03 (0.86, 1.22)	0.78	3% (0.39)
Non-selective beta-blocker	6883	53,870	215,978	1.09 (0.95, 1.25)	0.22	0% (0.47)	1.05 (0.92, 1.21)	0.45	0% (0.68)
All-cause mortality									
Medication use after diagnosis									
Main time-varying covariate analysis in stage 1–3 breast cancer patients									
Propranolol in stages 1–3^c^	17,219	96,097	382,1512	1.07 (0.93, 1.24)	0.32	29% (0.21)	1.13 (1.02, 1.24)	0.02	0% (0.46)
Main time-varying covariate analysis in all breast cancer patients									
Any beta-blocker	25,472	133,251	570,968	1.57 (1.41, 1.75)	<0.001	92% (<0.001)	1.12 (1.05, 1.20)	<0.001	65% (0.006)
Analysis based upon use in year after diagnosis^a^									
Propranolol	25,487	133,251	571,213	1.02 (0.89, 1.16)	0.82	48% (0.06)	1.04 (0.89, 1.21)	0.62	48% (0.06)
Non-selective beta-blocker	25,487	133,251	571,213	1.35 (1.17, 1.55)	<0.001	78% (<0.001)	1.14 (0.99, 1.30)	0.06	68% (0.003)
Medication use before diagnosis^b^									
Propranolol	31,556	139,760	664,448	0.97 (0.86, 1.09)	0.60	44% (0.09)	1.02 (0.94, 1.10)	0.68	0% (0.54)
Non-selective beta-blocker	31,556	139,760	664,448	1.30 (1.14, 1.49)	<0.001	80% (<0.001)	1.13 (1.06, 1.21)	<0.001	27% (0.21)

^a^Simplified analysis, not requiring time-varying covariate use, comparing medication users with non-users in the first year after diagnosis in individuals living more than 1 year after cancer diagnosis; fully adjusted column adjusted for age, year, stage and all confounders presented in Table 1

^b^Based on use in the year prior to diagnosis, restricted to individuals with 1 year of records prior to diagnosis; fully adjusted column only adjusted for age at diagnosis and year of diagnosis

^c^Excludes the Belgian cohort

*CI* confidence interval, *HR* hazard ratio

Table 4 also presents results for the analysis of propranolol use before diagnosis. Propranolol use in the year before diagnosis was not associated with reduced cancer-specific or all-cause mortality (fully adjusted HR = 1.03, 95% CI, 0.86, 1.22 and HR = 1.02, 95% CI, 0.94, 1.10, respectively). In all secondary analyses of non-selective beta-blocker use, similar associations were observed to those for propranolol use (see Table 4).

## Discussion

This large pooled analysis of breast cancer patients did not present convincing evidence of reduced cancer-specific or all-cause mortality in breast cancer patients who used propranolol or non-selective beta-blockers either before or after breast cancer diagnosis.

Our pooled analysis supports the findings of two earlier epidemiological studies of the association between propranolol use after diagnosis and cancer outcomes [15, 16]. The first, an earlier analysis of Danish data [16], showed no association between propranolol use after diagnosis and recurrence (adjusted HR = 1.3, 95% CI, 0.92, 1.9); however, that study did not investigate mortality or the influence of propranolol use before diagnosis. The second study, an earlier analysis of English data [15], based upon a case–control design, showed no association between propranolol and cancer-specific mortality (adjusted HR = 0.98, 95% CI, 0.57, 1.71).

Our pooled analysis also showed no reduction in cancer-specific mortality associated with propranolol use before diagnosis and therefore does not support the results of an earlier Irish study, the only previous study to investigate this association, which observed an 80% reduction in breast cancer-specific mortality (adjusted HR = 0.19, 95% CI, 0.06 0.60) in 46 breast cancer patients using propranolol in the year prior to diagnosis [14].

The main strength of our analysis is statistical power; this is the largest study yet to investigate the association between use of propranolol and cancer outcomes in breast cancer patients. Despite this, there remains the possibility of type 2 error and we cannot rule out a weak protective effect of propranolol on cancer-specific mortality. Other strengths include the long duration of follow-up, which was up to 13 years following breast cancer diagnosis in some cohorts. The use of routinely recorded drug information allowed precise evaluations of temporal relationships between propranolol use and mortality and eliminated the potential for recall bias incurred in questionnaire-based studies. Misclassification due to over-the-counter use was likely to be minimal because propranolol can be obtained only by prescription in the included countries.

A weakness of the study is the potential for bias due to the misclassification of breast cancer-specific cause of death on death certificates. However, simulations from a recent methodological study indicate that misclassification of breast cancer-specific cause of death is likely to have relatively small impact on comparisons between groups, assuming misclassification of cancer-specific death is not differential [40]. It should be noted that cohorts from three of the contributing countries [14–16] had been analysed previously with respect to propranolol; however, over 80% of the breast cancer patients included in the pooled analysis had not been analysed previously, and these earlier analyses covered different time periods [14, 16], were based on different study designs [14, 15], used a different outcome [16] or investigated only exposure before diagnosis [14]. There were some differences in the ascertainment of medication use (five studies used dispensing records, two used GP prescribing records and one used health insurance records) and in the ascertainment of mortality (seven studies used national mortality records and one used social security records). These differences may have contributed to the heterogeneity of the association between propranolol and all-cause mortality. This was partly due to the estimate in the Belgian cohort, and after removal of this study the heterogeneity was markedly reduced, but findings for all-cause mortality were similar. In contrast, there was little evidence of heterogeneity in the association between propranolol and cancer-specific mortality.

Oestrogen receptor status was not available in all of the cohorts; however, reanalysis of the propranolol association in the Swedish and Scottish cohorts additionally adjusting for oestrogen receptor status (after including tamoxifen and aromatase inhibitors in the model) made little difference to the estimates (data not shown), suggesting that oestrogen receptor status had limited potential to confound our results. BMI was also not available. The lack of adjustment for BMI could have attenuated propranolol associations because breast cancer patients with higher BMI have worse survival [41]. Similarly, we cannot rule out the effect of residual confounding on the observed associations from other unrecorded variables (such as trastuzumab use, diet, alcohol intake and physical activity) or for variables which were recorded differently between cohorts (such as use of hormone replacement therapy).

## Conclusions

In this large pooled analysis, propranolol and non-selective beta-blocker use, either before or after diagnosis, was not associated with improved breast cancer-specific or all-cause mortality.

## Abbreviations

CI: Confidence interval
GP: General practitioner
HR: Hazard ratio
SE: Standard error
